# EEG power at 3 months in infants at high familial risk for autism

**DOI:** 10.1186/s11689-017-9214-9

**Published:** 2017-09-13

**Authors:** April R. Levin, Kandice J. Varcin, Heather M. O’Leary, Helen Tager-Flusberg, Charles A. Nelson

**Affiliations:** 1 Boston Children’s Hospital, 300 Longwood Avenue, BCH 3213, Boston, MA 02115 USA; 2 Department of Neurology, Boston Children’s Hospital, Boston, MA USA; 3 Laboratories of Cognitive Neuroscience, Division of Developmental Medicine, Boston Children’s Hospital, Harvard Medical School, Boston, MA USA; 4 Telethon Kids Institute, University of Western Australia, Perth, WA Australia; 5 Department of Psychological and Brain Sciences, Boston University, Boston, MA USA; 6 Graduate School of Education, Harvard University, Cambridge, MA USA

**Keywords:** Autism, Electroencephalography, Early development, Infant siblings, Biomarker

## Abstract

**Background:**

Alterations in brain development during infancy may precede the behavioral manifestation of developmental disorders. Infants at increased risk for autism are also at increased risk for other developmental disorders, including, quite commonly, language disorders. Here we assess the extent to which electroencephalographic (EEG) differences in infants at high versus low familial risk for autism are present by 3 months of age, and elucidate the functional significance of EEG power at 3 months in predicting later development.

**Methods:**

EEG data were acquired at 3 months in infant siblings of children with autism (high risk; *n* = 29) and infant siblings of typically developing children (low risk; *n* = 19) as part of a prospective, longitudinal investigation. Development across multiple domains was assessed at 6, 9, 12, 18, 24, and 36 months. Diagnosis of autism was determined at 18–36 months. We assessed relationships between 3-month-olds’ frontal EEG power and autism risk, autism outcome, language development, and development in other domains.

**Results:**

Infants at high familial risk for autism had reduced frontal power at 3 months compared to infants at low familial risk for autism, across several frequency bands. Reduced frontal high-alpha power at 3 months was robustly associated with poorer expressive language at 12 months.

**Conclusions:**

Reduced frontal power at 3 months may indicate increased risk for reduced expressive language skills at 12 months. This finding aligns with prior studies suggesting reduced power is a marker for atypical brain function, and infants at familial risk for autism are also at increased risk for altered developmental functioning in non-autism-specific domains.

## Background

Autism spectrum disorder (ASD) is characterized by difficulties with social communication and the presence of restricted and repetitive behaviors or interests that emerge in the toddler years [1]. Infant siblings of children with ASD have a nearly 20% chance of developing ASD [2], a figure substantially higher than the estimated 1–2% prevalence in the general population [3]. This increased risk is not only specific to ASD, however. Among high-risk siblings who do not develop ASD, many demonstrate lower levels of verbal, nonverbal, and motor functioning than their low-risk counterparts [4–6].

Given increasing evidence for the efficacy of early intervention in improving developmental outcomes [7, 8], there is a need to delineate risk markers of atypical development to facilitate earlier intervention. Converging findings from prospective, longitudinal studies of high-risk infant siblings suggest that the first, overt behavioral signs of social communication delays associated with ASD do not manifest until late in the first year of life [9]. To assess even earlier risk signs for ASD and other developmental disorders, measuring the patterns of electrophysiological activity that ultimately determine or predict behavior offers significant promise. There is now accumulating evidence from electroencephalographic (EEG) studies suggesting that infants at high risk for ASD, regardless of whether they ultimately meet diagnostic criteria for ASD, have different developmental trajectories within the first year of life compared to low-risk infants [10–20]. For example, at 6 months of age, high-risk infants show reduced frontal EEG power across all frequency bands compared to low-risk infants [20]. How early in life these differences manifest between high- and low-risk infant siblings, and how specific these findings are in predicting particular developmental disorders, remains to be delineated.

In the current study, our primary aim was to determine (1) whether differences in baseline frontal EEG power between high- and low-risk infants are present by 3 months of age. To date, early electrophysiological markers before 6 months have been scarcely explored. Our secondary aims were to elucidate the functional significance of differences in EEG power in early development. In particular, we aimed to determine (2) whether EEG power at 3 months distinguishes infants later diagnosed with ASD and (3) whether EEG power at 3 months predicts development in other domains (e.g., language), particularly over the first year of life.

## Methods

### Study design and participants

Participants in the current study were part of a prospective investigation examining infants at high versus low familial risk for ASD across the first 3 years of life. Infants were classified as high risk for autism (HRA) if they had at least one older sibling with a community diagnosis of ASD. When possible, ASD diagnoses in older siblings were verified using both the Autism Diagnostic Observation Schedule (ADOS) [21] and the Social Communication Questionnaire (SCQ) [22] (*n* = 14, 56% of the final, included sample) with best estimate clinical judgment by a psychologist, where required. Five older siblings (20%) had their community diagnosis verified using the SCQ only as they did not have an ADOS assessment. Four older siblings (16%) did not have SCQ data and instead had their diagnosis verified using the ADOS only. Two HRA older siblings (8%) did not have an SCQ or ADOS and as such were unable to have their community diagnosis verified, although both had received their diagnoses in specialist ASD clinics. Infants were classified as low-risk controls (LRC) if they had a typically developing older sibling and no first- or second-degree family members with ASD. When possible, older siblings of LRC infants were screened with the ADOS and the SCQ (*n* = 8; 57% of the final included sample) to ensure they did not meet criteria for ASD. One LRC proband (7%) did not have an ADOS assessment though they did not meet criteria on the SCQ. One other LRC proband (7%) did not have SCQ data though did not meet criteria for ASD on the ADOS. Four LRC older siblings (29%) did not have an SCQ or ADOS; however, data from the infant siblings were included as their parents reported no clinical concerns in the proband. Inclusion criteria for infants included a gestational age of at least 36 weeks, no known prenatal or postnatal complications and no known genetic disorder.

EEG recordings were collected from 29 HRA infants (18 male) and 19 LRC infants (13 male) at 3 months of age. Of these, 25 HRA infants (14 male) and 14 LRC infants (9 male) provided adequate EEG data for processing, as described below, and were therefore included for analysis.

Final ASD outcomes for infants who had 3-month EEG recordings was determined on the basis of the ADOS using the revised algorithm in convergence with clinical best estimate at the infant’s most recent visit (either at 18, 24, or 36 months). Of the 25 HRA infants with adequate EEG data, 7 met criteria for ASD (HRA+; all based on 36 month assessment outcomes), 15 were classified as no ASD (HRA−; *n* = 1 based on 24-month assessment outcomes and *n* = 14 based on 36-month assessment outcomes), and 3 had not completed outcome visits at either 18, 24, or 36 months due to their discontinuation in the study prior to 18 months. Of the 14 LRC infants, 8 completed an outcome assessment and none met criteria for ASD (LRC−; *n* = 2 based on 18-month assessment outcomes; *n* = 1 based on 24-month assessment outcomes; *n* = 5 based on 36-month assessment outcomes). Six LRC infants did not have outcome assessments due to study funding ending prior to these children reaching 18 months of age. The median ADOS-2 comparison score (a metric ranging from 1 to 10 that quantifies ASD symptomatology on the basis of raw ADOS scores, with higher values representing greater symptom severity) [23] for the LRC group was 1 (interquartile range 1) and 2 (interquartile range 2.25) for the HRA group. For the outcome groups, the median ADOS-2 comparison scores were as follows: LRC− = 1 (interquartile range 1), HRA− = 1 (interquartile range 1), and HRA+ = 5 (interquartile range 3).

Institutional review board approval was obtained from Boston University and Boston Children’s Hospital (# X06-08-0374) prior to starting the study. Written, informed consent was obtained from all caregivers prior to their children’s participation in the study.

### Behavioral assessments

At 6, 12, 18, 24, and 36 months, infants were administered the Mullen Scales of Early Learning (MSEL) [24]. Age-standardized *t* scores were used to quantify development across five domains: gross motor (at 6 and 12 months only), fine motor, visual reception, expressive language, and receptive language.

The Autism Observation Scale for Infants (AOSI) [25] was administered at 9 and 12 months to quantify early markers associated with ASD. Higher scores indicate more atypical behaviors.

The ADOS was administered at 18, 24, and 36 months, along with clinical best estimate, to determine ASD diagnoses as described above.

### EEG acquisition

Infant baseline EEG data were acquired in a dimly lit, sound-attenuated, electrically shielded room. The caregiver was seated in a chair holding their infant. Continuous EEG was recorded for between 2 and 5 min. (The initial protocol included 2 min of recording, but this was later increased to 5 min to ensure that the majority of infants would have adequate data for analysis after exclusion of artifact-contaminated epochs, as described below. The recording was truncated prior to completion in infants who became excessively fussy). A research assistant sat to the right of the caregiver and the infant. They were in the room for the recording to assist in keeping the infant calm and still by blowing bubbles or presenting a quiet toy to the infant if they became fussy (e.g., a ball).

EEG data were collected using either a 64-channel Geodesic Sensor Net System or a 128-channel Hydrocel Geodesic Sensor Net System and a Net Amps 200 amplifier or a Net Amps 300 high-input amplifier (Electrical Geodesics, Inc., Eugene, OR, USA). Both systems and net type were used across both risk groups. The majority of infants, however, had EEG recorded using 128-channel nets on a Net Amps 300 system (68% HRA; 86% LRC). Data were sampled at either 250 or 500 Hz and referenced to the vertex (electrode Cz), with impedances kept below 100 kΩ (within recommended guidelines given the high-input impedance capabilities of this system’s amplifier). Electrooculographic electrodes were removed to enhance infants’ tolerance of the net.

### EEG pre-processing

Initial pre-processing of data took place in Net Station (EGI, Inc., Eugene, OR). A 1-Hz high-pass filter and a 60-Hz notch filter were applied. Any channel with excessive artifact in the majority of the recording (such that leaving that channel in the analysis would result in significantly more epoch exclusions, as described below, than interpolating that channel) and any channel that did not contain EEG data based on visual inspection of the signal (e.g., channels with flat tracings, or those in which low-frequency activity generally looked dissimilar to that of surrounding electrodes) were marked for interpolation. The number of interpolated channels did not exceed 10% of total active electrodes for any infant. Data were re-referenced to average after excluding marked channels (and later also transformed using a Laplacian reference, as described below). Data from all channels, including interpolated channels, was then exported to MATLAB (R2015a). In MATLAB, data sampled at 500 Hz were low pass filtered with a cutoff frequency of 100 Hz and downsampled to 250 Hz. Data were detrended using a Kalman filter [26] (*b* = 0.995) and epochs with high-amplitude artifact (> 150 μV) in any channel were excluded from further analysis. Remaining segments of useable data were then further segmented into one-second, non-overlapping segments. Infants with fewer than 10 good segments were excluded from further analyses.

All analyses were initially run with average referencing and then repeated with a Laplacian reference, as Laplacian referencing has been shown to reduce sensitivity of the EEG signal to contamination by myogenic activity, particularly in central regions but also in frontal and occipital regions [27]. Laplacian referencing involves referencing the activity in each electrode to that of its nearest neighbors, thus enhancing sensitivity to local activity and reducing sensitivity to more diffuse or volume-conducted activity.

### EEG power analysis

A fast Fourier transform with a Hanning window was used to calculate a power spectrum on each segment. For each EEG, the average power spectrum across all one-second segments was then calculated. As our primary aim involved determining if differences in *frontal* EEG power previously reported among high-risk infants at 6 months [20] extend to earlier in life (i.e., at 3 months), our primary region of interest was over the frontal region. We replicated a previously described frontal region of interest, [20] centered on electrodes F3 and F4. In the 64-channel net, this frontal region included channels 3, 8, 9, 13 (F3), 16, 57, 58, and 62 (F4); in the 128-channel net, it included channels 3, 4, 10, 18, 19, 20, 23, 24 (F3), 27, 118, 123, and 124 (F4). Because the Laplacian-referenced data was calculated only on electrodes in the 10–20 electrode reference system (using surrounding electrodes in the high-density array as the reference electrodes), the frontal region for the Laplacian reference included channels F3 and F4. A spectral slope between 20 and 200 Hz of greater than − 0.1 has previously been used to suggest significant contribution from muscle artifact [28], since the EEG power spectrum from brain activity generally fits a 1/(*f* ^*α*^) (“pink noise”) structure. Because our analyses evaluated power only up to 50 Hz, and because our sampling rates would result in 200 Hz being well above the Nyquist frequency for many of the EEGs included in this analysis, we measured slope of the power spectrum between 20 and 50 Hz. These measurements were conducted on a log-log plot by using the MATLAB *polyfit* function.

### Statistical analyses

Results are presented two ways: Binned by frequency band, and unbinned. Binned power is more standard in the developmental literature and allows for assessment of oscillatory activity that rises above background activity [29]. However, recent evidence suggests that the background activity itself (i.e., the “shape” of the power spectrum) also contains crucial information about underlying electrophysiology [30, 31]. Unbinned power allows assessment of overall trends in this background activity.

For analysis of binned data, frequency bands were defined as previously described (delta [2–4 Hz], theta [4–6 Hz], low alpha [6–9 Hz], high alpha [9–13 Hz], beta [13–30 Hz], gamma [30–50 Hz]) [20]. Here we report all binned data as absolute power values, normalized by a log 10 transform. Behavioral data and EEG data in some frequency bands were non-normal (based on a Shapiro-Wilk, *p* < .05 and histogram observation). As such, Mann-Whitney *U* nonparametric tests were performed to compare binned power values and performance on behavioral tasks between HRA and LRC groups. A Kruskal-Wallis one-way analysis of variance was performed to compare binned power values between ASD outcome groups (LRC−, HRA+, HRA−). Spearman’s rho correlations were performed to examine associations between binned frontal power values at 3 months with developmental functioning on the MSEL and AOSI. In this regard, we primarily focused on the association between 3-month frontal EEG power and developmental functioning on the MSEL and AOSI over the first year of life (i.e., at 6–12 months). However, in order to examine the persistence (or transience) of any significant associations over the first year, we then conducted a separate set of analyses focused on the association between 3-month frontal EEG power and MSEL scores over the second and third years of life (i.e., at 18, 24, and 36 months). Of note, power to detect such associations on the MSEL was higher in the first year of life due to a decline in sample size at later ages (6 months: *n* = 36; 12 months: *n* = 36; 18 months: *n* = 30; 24 months: *n* = 28; 36 months: *n* = 26). A false discovery rate (FDR) correction at *p* < .05 was applied to the *p* values from nonparametric tests to control for multiple comparisons [32]. For binned power analyses, the FDR correction was applied separately to average reference power data and Laplacian-transformed data, and to analyses focused on 6–12- and 18–36-month outcomes. All reports of significance for binned data are with reference to those effects that remained significant after FDR correction. All of the abovementioned analyses were conducted using SPSS version 23.0 (IBM Corp, Armonk, NY).

For analysis of unbinned data, we computed the median and 25th–75th percentile at each point of the power spectrum within each group, as well as the median difference in group-averaged power spectra by using a frequency domain-based bootstrapping algorithm with 2000 replications, as previously described, [33, 34] using publicly available MATLAB code [33]. Because these analyses in their current form can only be used to compare outcomes between two groups, analyses of unbinned data were restricted to HRA versus LRC, and HRA+ versus HRA−.

## Results

Of note, the final LRC and HRA samples did not differ significantly in their gender distribution (*X* ^2^ = .26, df = 1, *p* = .614 [LRC: *n* = 5/14, 36% female; HRA: *n* = 11/25, 44% female] or age at the time of their EEG recording (*t*(35) = 1.71, *p* = .097 [LRC mean age = 3.68 months, SD = .44; HRA mean age = 3.41 months, SD = .49]).

### Behavioral assessments

#### Mullen Scales of Early Learning

##### Risk group (HRA/LRC) comparison

*6 and 12 months*: At 6 months of age, there was a trend toward significantly lower gross motor scores in the HRA group compared to the LRC group (Table 1), *U* = 87.50, *z* = − 1.925, *p* = .057. There were no differences between risk groups in any other subdomains (all *p* values > .05). At 12 months, the HRA group revealed a trend toward lower gross motor scores, *U* = 94.00, *z* = −1.801, *p* = .077, and significantly lower scores in the expressive language domain, *U* = 65.50, *z* = − 2.913, *p* = .003. There were no group differences in visual reception, fine motor, or receptive language skills at 12 months (all *p* values > .05). Median subscale scores (and interquartile ranges) for each risk group are presented in Table 1.

**Table 1:** Risk group (HRA, LRC) developmental functioning scores at 6, 9, 12, 18, 24, and 36 months

	Risk groups											
	6 months		9 months		12 months		18 months		24 months		36 months	
	HRA (*n* = 22)	LRC (*n* = 14)	HRA (*n* = 14)	LRC (*n* = 11)	HRA (*n* = 22)	LRC (*n* = 14)	HRA (*n* = 21)	LRC (*n* = 9)	HRA (*n* = 22)	LRC (*n* = 6)	HRA (*n* = 21)	LRC (*n* = 5)
Gross motor	46 (13)	53 (12)	–	–	43 (20)	48 (23)	–	–	–	–	–	–
Fine motor	46 (12)	46 (9)	–	–	60 (21)	60 (12)	54 (10)	49 (14)	48 (8)	50 (17)	45 (15)	55 (33)
Visual reception	45 (13)	45 (19)	–	–	54 (11)	58 (10)	48 (7)	56 (10)	49 (10)	56 (13)	58 (23)	58 (24)
Expressive language	45 (8)	45 (6)	–	–	46 (10)	57 (7)	51 (13)	54 (7)	54 (17)	58 (8)	56 (14)	56 (16)
Receptive language	47 (9)	44 (2)	–	–	44 (10)	44 (5)	55 (30)	69 (11)	53 (8)	64 (10)	54 (12)	58 (17)
AOSI total	–	–	4 (5)	5 (4)	3 (3)^a^	3 (5)^b^	–	–	–	–	–	–

Median scores (and interquartile range) on developmental assessments for infants with EEG data at 3 months

^a^One HRA infant was missing AOSI data at 12 months

^b^6 LRC infants were missing AOSI data at 12 months

*18, 24, and 36 months*: At 18 months, the HRA group had significantly lower visual reception, *U* = 40.00, *z* = −2.501, *p* = .012, and receptive language scores, *U* = 33.00, *z* = − 2.798, *p* = .004 compared to the LRC group. These differences persisted at 24 months, with the HRA group demonstrating significantly lower visual reception scores, *U* = 25.50, *z* = − 2.292, *p* = .020, and receptive language scores, *U* = 20.00, *z* = − 2.594, *p* = .008, compared to the LRC group. There were no group differences in any other subdomains at 18 or 24 months. There were no group differences in any subscale at 36 months (all *p* values > .05). Median subscale scores (and interquartile ranges) are presented in Table 1.

##### Outcome group (HRA+/HRA−/LRC−) comparison

*6 and 12 months*: A Kruskal-Wallis *H* test found no significant differences between outcome groups on any subscales of the Mullen at 6 months of age (all *p* values > .05). At 12 months, however, there were significant group differences in the receptive language (*H*(2) = 9.87, *p* = .007) and expressive language (*H*(2) = 9.59, *p* = .008) subscales. Follow-up pairwise comparisons showed that the HRA+ group at 12 months had significantly lower receptive language scores compared to the HRA− group (*p* = .005) and significantly lower expressive language scores compared to the LRC− group (*p* = .006). There were no group differences in visual reception or fine motor skills at 12 months (all *p* values > .05). Median subscale scores (and interquartile ranges) for each outcome group are presented in Table 2.

**Table 2:** Outcome group (HRA+, HRA−, LRC−) developmental functioning scores at 6, 9, 12, 18, 24, and 36 months

	Outcome groups																	
	6 months			9 months			12 months			18 months			24 months			36 months		
	HRA+ (*n* = 7)	HRA− (*n* = 14)	LRC− (*n* = 8)	HRA+ (*n* = 6)	HRA− (*n* = 11)	LRC− (*n* = 5)	HRA+ (*n* = 7)	HRA− (*n* = 14)	LRC− (*n* = 8)	HRA+ (*n* = 6)	HRA− (*n* = 15)	LRC− (*n* = 8)	HRA+ (*n* = 7)	HRA− (*n* = 15)	LRC− (*n* = 6)	HRA+ (*n* = 7)	HRA− (*n* = 14)	LRC− (*n* = 5)
Gross motor	40 (13)	47 (9)	53 (10)	–	–	–	43 (17)	40 (23)	48 (20)	–	–	–	–	–	–	–	–	–
Fine motor	46 (12)	46 (14)	46 (6)	–	–	–	55 (19)	62.5 (16)	57.5 (15)	53 (6)	54 (14)	52 (17)	48 (10)	48 (8)	50 (17)	39 (17)	50 (14)	55 (33)
Visual reception	45 (7)	45 (19)	45 (18)	–	–	–	49 (9)	57 (9)	60 (8)	48 (18)	48 (6)	56 (11)	49 (9)	53 (10)	56 (13)	52 (25)	59 (23)	58 (24)
Expressive language	48 (6)	42 (8)	48 (6)	–	–	–	37 (8)	46 (9)	54 (6)	42 (17)	51 (10)	54 (9)	41 (28)	56 (15)	58 (8)	49 (16)	57 (11)	56 (16)
Receptive language	44 (6)	47 (19)	44 (0)	–	–	–	37.5 (11)	44 (6)	44 (5)	33 (33)	59 (22)	70 (8)	50 (26)	56 (7)	64 (10)	49 (7)	56 (9)	58 (17)
AOSI total	–	–	–	3 (5)	4 (6)	3 (5)	4 (3)	2.5 (3)	1.5 (3)	–	–	–	–	–	–	–	–	–

Median scores (and interquartile range) on developmental assessments for infants with EEG data at 3 months

*18, 24, and 36 months*: A Kruskal-Wallis *H* test found significant differences between outcome groups at both 18 and 24 months. Specifically, at 18 months, there were significant differences in the visual reception (*H*(2) = 6.47, *p* = .039), receptive language (*H*(2) = 12.55, *p* = .002), and expressive language (*H*(2) = 6.26, *p* = .044) subscales. Follow-up pairwise comparisons showed that for the visual reception and expressive language subscales, there was a trend for the HRA+ group to have the lowest scores; however, no comparisons reached significance. For the receptive language subscale, follow-up pairwise comparisons showed that both the HRA+ and the HRA− groups had significantly lower scores compared to the LRC− group at 18 months (*p* = .002 and *p* = .041, respectively). At 24 months, there were significant differences in the visual reception (*H*(2) = 6.41, *p* = .041) and receptive language (*H*(2) = 14.54, *p* = .001) subscales. For the visual reception subscale, follow-up pairwise comparisons showed that the HRA+ group had significantly lower scores compared to the LRC− group at 24 months (*p* = .037). For the receptive language subscale, the HRA+ group had lower scores than both the HRA− group (*p* = .016) and the LRC− group (*p* = .001). There were no outcome group differences in any subscale at 36 months (all *p* values > .05). Median scores are presented in Table 2.

### Autism Observation Scale for Infants

Of note, only a subset of the sample had AOSI data available at 9 and 12 months (9 months: 56% of the HRA group, 70% of the LRC group; 12 months: 84% of the HRA group, 57% of the LRC sample).

#### Risk group (HRA/LRC) comparison

There were no significant differences in total scores between HRA and LRC infants at 9 months on the AOSI (*U* = 101.50, *z* = − .130, *p* = .899) or 12 months (*U* = 165.50, *z* = .377, *p* = .713). Medians and interquartile ranges for the total scores at 9 and 12 months (for the final, included sample of infants with good EEG data) are reported in Table 1.

#### Outcome group (HRA+/HRA−/LRC−) comparison

There were no significant differences in total scores between the outcome groups on the AOSI at 9 months (*H*(2) = .33, *p* = .849) or 12 months (*H*(2) = 4.18, *p* = .123). Medians and interquartile ranges are reported by outcome group in Table 2.

### EEG data quality

There were no differences in power as a function of net and amplifier type within any frequency band, in either the average referenced data (consistent with findings from Tierney et al.) or Laplacian-transformed data; all *p* values > .05. As such, all further analyses were conducted with EEG data collapsed across acquisition setups. There were no significant group differences in the number of interpolated channels between the HRA (median = 2 channels) and the LRC (median = 2 channels) groups, *U* = 183.50, *z* = .256, *p* = .806, or between LRC− (median = 1.5 channels), HRA− (median = 2 channels), and HRA+ (median = 1 channel) infants, *H*(2) = 3.53, *p* = .171. The median number of good segments did not significantly differ between the HRA (media*n* = 73) and the LRC (median = 57.5) groups, *U* = 206.50, *z* = .922, *p* = .361. There were also no significant differences in the number of good segments between LRC− (median = 79), HRA− (media*n* = 60), and HRA+ (median = 70), *H*(2) = .114, *p* = .945). We also calculated the proportion of data retained (versus rejected) after artifact detection as a function of the total EEG recording length. The proportion of good data within each recording did not significantly differ between HRA (median 45.34%) and LRC groups (median 41.95%), *U* = 209.00, *z* = .995, *p* = .331, or LRC− (median: 50.18%), HRA− (45.34%), and HRA+ (43.19%) groups, *H*(2) = .261, *p* = .877. Spectral slope met the aforementioned criteria of being <− 0.1 for all groups, across all channels standardly included in a 10–20 system.

### Power spectral density at 3 months by ASD risk status: HRA versus LRC

**Fig. 1:**
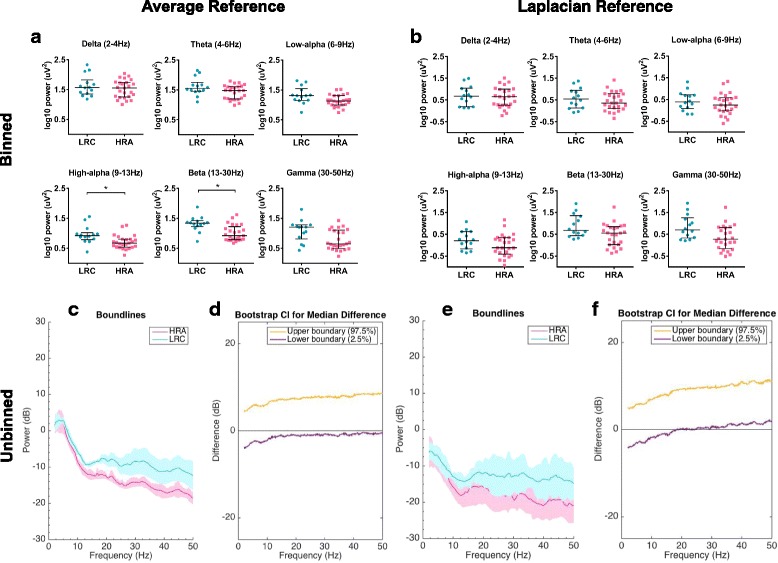
3-month frontal EEG power by risk group. **a**, **b** Frontal binned power spectral density (log10 transformed) at 3 months of age for LRC and HRA risk groups for each frequency band, using **a** average-referenced data and **b** Laplacian-referenced data. Asterisks (*) denote significant results after FDR correction. **c** Frontal unbinned group-median power spectra (solid line, median; shaded area, 25th–75th percentile) showing EEG power at 3 months of age across all frequencies in HRA and LRC groups, for average-referenced data. (Pink line, HRA; cyan line, LRC). **d** Differences in frontal unbinned group-median power spectra at 3 months of age, presented with 95% CI from the bootstrap analysis (average-referenced data) (purple line, 2.5th percentile; orange line, 97.5th percentile). EEG power is similar across all frequencies for the HRA as compared to the LRC group. **e**, **f** Same analysis as (**c**, **d**), for Laplacian-referenced data. A significant decrease in frontal power in the HRA group compared to the LRC group emerges above approximately 20 Hz. LRC = low-risk control; HRA = high-risk for autism

*Average reference, binned analyses*: In the binned average-referenced data, the HRA group had significantly lower power over the frontal region in the high-alpha band (*U* = 71.00, *z* = −3.045, *p* = .002, *r* = .49) and beta band (*U* = 70.00, *z* = −3.074, *p* = .002, *r* = .49) after the FDR correction (Fig. 1a). There were no significant group differences in any other frequency band over the frontal region.

*Average reference, unbinned analyses*: In the unbinned average-referenced data, there were no significant differences in group-averaged frontal (Fig. 1c, d) power spectra between HRA and LRC groups.

*Laplacian reference, binned analyses*: In the binned Laplacian-referenced data, there were no significant differences between HRA and LRC groups in any frequency band over the frontal (Fig. 1b) region.

*Laplacian reference, unbinned analyses*: In the unbinned Laplacian-referenced data, the HRA group had significantly lower power over the frontal region in all frequencies greater than 20 Hz, as compared to the LRC group (Fig. 1e, f).

### Power spectral density at 3 months by ASD outcome: LRC−, HRA−, and HRA+

**Fig. 2:**
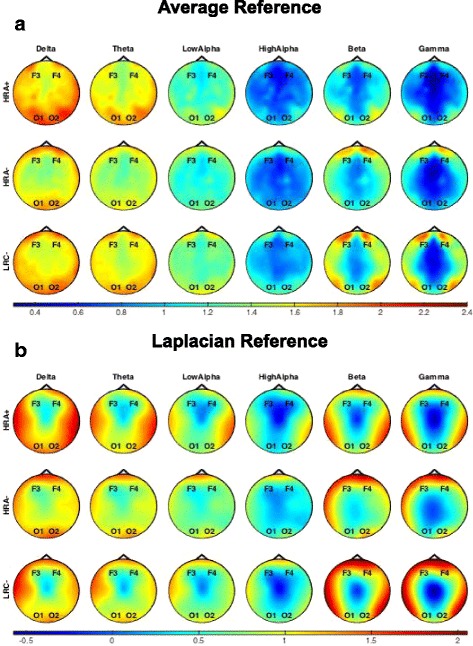
Topoplots demonstrating the scalp distribution of EEG power for **a** average-referenced and **b** Laplacian-referenced data. Rim electrodes not shown. For each frequency band, plots represent average power for HRA+, HRA−, or LRC− groups. Color bars represent log10 transformed power

Topoplots demonstrating the distribution of power across the scalp in each frequency band are provided in Fig. 2.

**Fig. 3:**
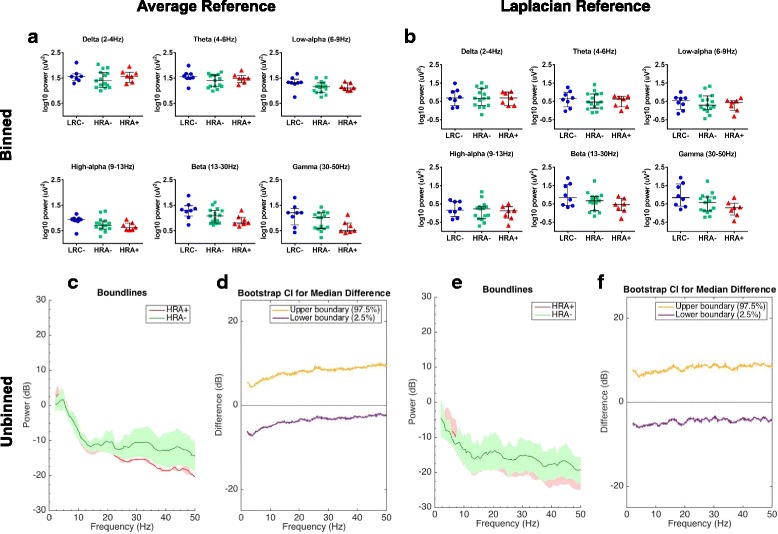
3-month frontal EEG power by outcome group. **a**, **b** Frontal binned power spectral density (log10 transformed) at 3 months of age for LRC−, HRA−, and HRA+ outcome groups for each frequency band, using **a** average-referenced data and **b** Laplacian-referenced data. **c** Frontal unbinned group-median power spectra (solid line, median; shaded area, 25th–75th percentile) showing EEG power at 3 months of age across all frequencies in HRA+ and HRA− groups, for average-referenced data. (Red line, HRA+; green line, HRA−). **d** Differences in frontal unbinned group-median power spectra at 3 months of age, presented with 95% CI from the bootstrap analysis (average-referenced data) (purple line, 2.5th percentile; orange line, 97.5th percentile). EEG power is similar across all frequencies for the HRA+ as compared to the HRA− group. **e**, **f** Same analysis as (**c**, **d**), for Laplacian-referenced data. There are no significant differences in power in the HRA+ group compared to the HRA− group at any frequency. LRC− = low-risk control, no ASD; HRA− = high-risk for autism, no ASD; HRA+ = high-risk for autism, with ASD

*Average reference, binned analyses*: We conducted a Kruskal-Wallis *H* test to examine differences in power between outcome groups (LRC−, HRA−, HRA+). While binned analyses for the average-referenced data revealed a pattern of lower frontal power through higher frequency bands (high-alpha, beta, gamma) among HRA+ infants, no effects reached statistical significance in our sample (Fig. 3a).

*Average reference, unbinned analyses*: Unbinned analyses for average referenced data revealed no significant differences across the power spectrum in HRA+ infants as compared to HRA− infants (Fig. 3c, d).

*Laplacian reference, binned analyses*: There were no significant differences as a function of outcome group in any frequency band for Laplacian-referenced power data (all *p* values > .05) (Fig. 3b).

*Laplacian reference, unbinned analyses*: Unbinned analyses for Laplacian-referenced data revealed no significant differences across the power spectrum in HRA+ infants as compared to HRA− infants (Fig. 3e, f).

### Correlations between 3-month frontal EEG power and developmental functioning

#### Autism Observation Scale for Infants

There were no significant correlations between EEG power at 3 months (in either average-referenced data or Laplacian transformed) and AOSI total scores at either 9 or 12 months (all *p* values > .05).

#### Mullen Scales of Early Learning

**Fig. 4:**
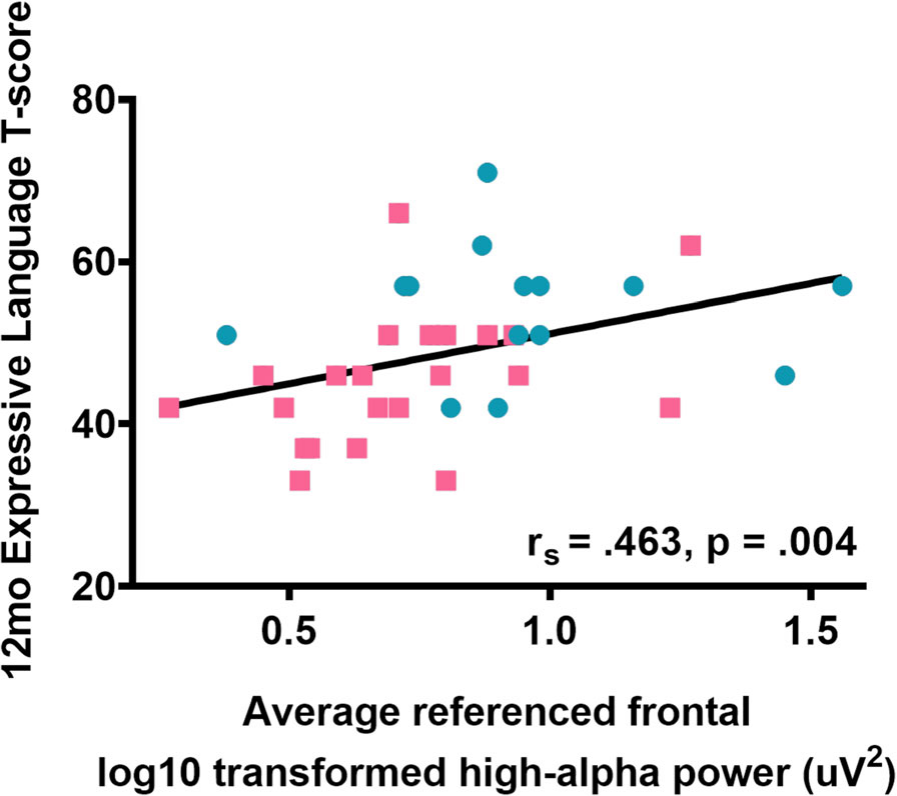
Spearman’s rho correlations (with fitted line) between 3-month frontal power (log10 transformed) in the high-alpha (9–13 Hz) band and *T* scores on the MSEL expressive language subscale for LRC (cyan circles) and HRA (pink squares) infants

*6 and 12 months*: For the average-referenced power data, there was a positive correlation between 3-month frontal high-alpha power and expressive language level at 12 months (*r*_s_ = .463, *N =* 36, *p* = .004) that survived FDR correction (Fig. 4). No other correlations between 3-month frontal EEG power and developmental functioning, on any subscale, survived correction for multiple comparisons. For the Laplacian-transformed power, there were no significant correlations between 3-month frontal power and developmental functioning either prior, or subsequent, to FDR correction (all *p* values > .05).

*18, 24, and 36 months*: There were no significant correlations between 3-month frontal EEG power and scores on the MSEL subscales that survived FDR correction at 18, 24, or 36 months for either the average-referenced power or Laplacian-transformed power.

## Discussion

The findings reported here reveal that 3-month-old infants at high familial risk for ASD have reduced frontal high alpha and beta power (in an average-referenced, binned analysis) and reduced frontal power in frequencies above 20 Hz (in a Laplacian-referenced, unbinned analysis) compared to same-aged infants at low familial risk for ASD. Reduced frontal power did not predict ASD-specific outcomes; however, reduced frontal high-alpha power at 3 months was associated with reduced expressive language skills at 12 months.

A key finding here is the early age (3 months) at which EEG findings correlate with risk status, and with later developmental outcomes. To our knowledge, while decreased EEG power in young children has been previously associated with increased risk for social and communication difficulties [20, 35], no study has previously evaluated the association between EEG power and developmental risk or outcome in infants younger than 6 months. Our findings are consistent with prior studies suggesting that physiologic changes may be detectable before behavioral changes become manifest in a variety of neurological and neurodevelopmental disorders [36–43].

Notably, the association between 3-month frontal high-alpha power and expressive language skills appeared to be developmentally time-locked to the 12-month time point, with no evidence of this association persisting over the second and third years of life in our sample. This can be interpreted in one of two ways. First, 3-month power may portend only a transient decrease in expressive language skills, which resolves by 18–36 months of age. Alternatively, it is important to note that the sample size for participants who underwent MSEL testing decreases from 1 to 3 years of age in our study. Therefore, it is worth considering the possibility that a larger sample size at 18-, 24-, and 36-month time points would have been better powered to identify any persistent correlations between 3-month frontal high-alpha power and later expressive language skills.

A second key finding is that reduced EEG power at 3 months correlates most robustly with familial ASD risk and at least a transient change in expressive language development, rather than ASD outcomes specifically. This is consistent with studies suggesting that infants carrying a familial high-risk ASD endophenotype can present with altered development in multiple domains [4, 6]. Thus while ASD and language delay are considered to be discrete (albeit frequently overlapping) clinical entities, the physiology (as measured by EEG) is consistent with prior studies suggesting that the underlying risk factors for these disorders may overlap [44]. For example, alpha oscillations have been associated with temporal integration [45, 46], attentional control of speech processing [47], and verbal fluency [48]. Frontal alpha power may thus predict later expressive language by altering an infant’s ability to attend to, integrate, and ultimately produce speech; however, it may also affect an infant’s ability to attend to and integrate nonverbal stimuli as well.

While these associations are noteworthy, we cannot delineate the physiologic mechanisms underlying low frontal EEG power in high-risk infants from these findings alone. This is an important avenue for future research. In particular, contributions of muscle activity are difficult to quantify in infants, and the awake infant EEG signal will include a combination of information about underlying brain activity and muscle or other artifact. We took numerous steps to limit the impact of such artifact on our data, including the use of the Laplacian reference, [27] conservative artifact removal in which epochs with even one channel with high-amplitude artifact were removed from further analysis, verification that the slope of the power spectra was always <− 0.1, consistent with brain activity more so than muscle activity [28], and verification that measures of EEG data quality did not differ between groups. While these steps serve to enhance the signal to noise ratio of the EEG, we maintain that the contribution of muscle and other artifact cannot be entirely disentangled and, as such, the findings should be considered in this context.

The different findings seen across referencing types also merit further discussion here, because the sum of these complementary analyses provides a more nuanced picture of brain function than any single analysis alone. First, presenting analyses using both average referencing and Laplacian referencing allows for more targeted analysis of widespread versus localized activity. Our results demonstrate that the increased activity in high-alpha and beta in the HRA group (compared to LRC) and the correlation between high-alpha and expressive language are only present in the average-referenced binned analyses; they are not present in the Laplacian-referenced binned analyses. By referencing the EEG tracing at a given electrode to that of its nearest neighbors, the Laplacian reference mitigates the effect of diffuse activity sensed by multiple electrodes (including volume-conducted artifact but also widespread neural activity) and improves sensitivity to local activity. One possibility is therefore that our average-referenced findings are due to volume-conducted artifact and thus removed by the Laplacian reference. Alternatively, it remains quite possible that the relevant high-alpha oscillations are true neural activity, but are diffuse rather than localized, and are therefore removed by Laplacian referencing.

Additionally, binned and unbinned analyses provide complementary information. While binned analyses allow for assessment of oscillatory activity that rises above background activity [29], recent evidence suggests that the shape of the power spectrum, best measured by unbinned analyses, also contains highly relevant information about underlying physiology. Our unbinned Laplacian analyses suggest that the shape of the power spectrum is different between HRA and LRC groups, with the HRA group displaying decreased power above 20 Hz. This decreased power in higher frequencies can be seen when muscle activity is decreased (as above), but also when spontaneous background neuronal firing activity is decreased [30, 31]. This activity can be altered by abnormal baseline neuronal function (e.g., if neurons in HRA infants are too “passive” without adequate spontaneous neural activity). It has been hypothesized, for example, that spontaneous background neural “noise” is necessary to prevent excessive local overcoupling [31]. However, such activity can also be altered by behavioral or cognitive state. Therefore, one cannot rule out the possibility that differences in EEG power may be due to differences in infant state or behavior during data acquisition. Notably, however, these two possibilities (altered neuronal function and altered state) are not mutually exclusive; in fact, they are highly interrelated. Specifically, if background neuronal activity is altered in the HRA group, this may also alter the infant’s tendency toward a particular behavioral state during the baseline EEG collection paradigm.

Taken in combination with other studies evaluating brain rhythms in high-risk and low-risk groups at different times during development, the findings described here emphasize the dynamic nature of development. At 3 months, we see reduced average-referenced, binned frontal power in high-risk infants only in the high alpha and beta bands, whereas a prior study suggested that by 6 months, this pattern is seen across all frequency bands [20]. Of course, caution should be exercised in comparing findings across studies, given differences in processing techniques. (For example, in order to avoid concatenation of epochs otherwise interrupted by artifact, as this can introduce spurious findings at the boundaries between epochs, detrending and windowing settings in the current study are from those described in Tierney et al.). Even so, the possibility that diffuse low power in the high alpha and beta bands could portend developmental profiles of atypical oscillations in other frequency bands over the next several months is interesting to consider, particularly in the context of other studies suggesting altered trajectories of functional connectivity in high risk infants [17, 49]. Future studies examining trajectory of neural rhythms over time in high-risk and low-risk groups, beginning in early infancy and including large sample sizes into the third year of life and beyond, will be of tremendous benefit in improving our understanding of such trajectories and their implications.

## Conclusions

Overall, the findings described here suggest that frontal EEG power at 3 months of age differs in infants at high versus low risk for ASD, and correlates with development of expressive language skills at 12 months of age. This work aligns with an emerging body of evidence demonstrating changes in brain development prior to the manifestation of overt behavioral changes in infants at high familial risk for ASD [39, 42, 43], and points to the potential of brain-based markers for early identification and prognostication in neurodevelopmental disorders. Future studies will offer the opportunity to elucidate the mechanisms underlying this finding, to better characterize the long-term trajectories of brain development that underlie behavior, and to determine potential clinical implications of these findings.

## Abbreviations

ADOS: Autism Diagnostic Observation Schedule
AOSI: Autism Observation Scale for Infants
ASD: Autism spectrum disorder
EEG: Electroencephalography/electroencephalographic
FDR: False discovery rate
HRA: High risk for autism
HRA-: High-risk infants without autism
HRA+: High-risk infants with autism
LRC: Low-risk controls
LRC-: Low-risk controls without autism
MSEL: Mullen Scales of Early Learning
SCQ: Social Communication Questionnaire
